# The continuous net benefit: assessing the clinical utility of prediction models when informing a continuum of decisions

**DOI:** 10.1186/s41512-026-00224-z

**Published:** 2026-02-17

**Authors:** Jose Benitez-Aurioles, Laure Wynants, Niels Peek, Patrick Goodley, Philip Crosbie, Matthew Sperrin

**Affiliations:** 1 Centre for Health Informatics, University of Manchester, Vaughan House, Portsmouth Street, Manchester, M13 9GB UK; 2 Department of Epidemiology, Care and Public Health Research Institute, Maastricht University, Maastricht, The Netherlands; 3 Department of Development and Regeneration, KU Leuven, Leuven, Belgium; 4 Leuven Unit for Health Technology Assessment Research (LUHTAR), Department of Public Health and Primary Care, KU Leuven, Leuven, Belgium; 5 THIS Institute, University of Cambridge, Cambridge, UK; 6 Division of Immunology, Immunity to Infection & Respiratory Medicine, School of Biological Sciences, University of Manchester, Manchester, UK; 7 Manchester Thoracic Oncology Centre, Manchester University NHS Foundation Trust, Manchester, UK

**Keywords:** Clinical prediction models, Net benefit, Decision curve analysis, Prognosis

## Abstract

**Background:**

The net benefit and decision curve analysis are increasingly being used to assess the clinical utility of prognostic models. This metric assesses the value added by a model’s predictions when individuals are treated differently according to whether they are over or under a chosen threshold. Although such ‘treat or not’ decisions are common, prognostic models are also often used to tailor and personalise the care of patients, which implicitly involves the consideration of multiple interventions at different risk thresholds. We aim to extend decision curve analysis to estimate the net benefit of a model over multiple thresholds.

**Methods:**

We take a weighted area under a rescaled version of the net benefit curve, deriving the continuous net benefit. In addition to the consideration of a continuum of interventions, we also show how the continuous net benefit can be used to evaluate single treatments in populations with a range of optimal thresholds, due to individual variations in expected treatment benefit or harm, highlighting limitations of current proposed methods that calculate the area under the decision curve. We propose this not as a substitute for decision curves, but as a complementary evaluation metric, in lieu of single-threshold point estimates.

**Results:**

We showcase this metric through two examples of model validation in cardiovascular preventive care. The continuous net benefit brought additional insight over point estimates when comparing models over a range of decisions.

**Conclusions:**

The continuous net benefit informs those looking to validate clinical prediction models of their clinical utility, and helps decision makers understand their usefulness, improving their viability towards implementation.

**Supplementary Information:**

The online version contains supplementary material available at 10.1186/s41512-026-00224-z.

## Introduction

Clinical prediction models are used to determine a patient’s diagnostic or prognostic risk. This can be useful when deciding whether patients should be screened, treated, monitored, or referred for diagnosis. Performance metrics such as the C-statistic or calibration plots can be used to assess the performance of these models irrespective of the clinical context and decisions that the model is meant to support [1, 2], but are insufficient when determining if they will be useful in practice [1, 3]. 

Decision curve analysis is used to compare the clinical utility of such models using the net benefit [4]. Models are evaluated by binarizing the risk scores at a clinically informed choice of threshold [5], with the decision curve plotting this threshold-specific net benefit for a range of thresholds. Originally designed for models informing a single clinical decision [4], a model’s output is often not used just to answer a single ‘treat or not’ question. For example, in cardiovascular disease prevention, a clinician might use QRISK to decide whether to give a patient lifestyle recommendations, refer them to a smoking cessation programme, prescribe statins and/or antihypertensives, and increase monitoring frequency [6–9]. Within the same clinical setting, thresholds can also differ between patients because of differences in expected treatment benefit or potential harm. For instance, individuals with liver disease might be cautioned against taking statins and therefore require a higher cardiovascular risk before treatment is considered appropriate. In addition, even among clinically similar patients, thresholds may vary because of personal attitudes towards risk. In shared decision-making settings, some patients may prefer to defer treatment until their estimated risk is markedly higher after discussing possible complications with their clinician [10]. Reporting the benefit of a model for a single threshold would not reflect the true threshold variability, would misrepresent the underlying uncertainty, and would overestimate precision. In such cases, a model is considered superior to another model if its corresponding decision curve is above the other model’s curve over the entire range of relevant thresholds. Whilst you can interpret a difference in net benefit between two models for a particular threshold, you cannot meaningfully interpret the difference in net benefit of one model over many thresholds. This introduces difficulties in developing metrics that ‘summarize’ the net benefit over a range, and these approaches are subject to debate. However, a single-metric evaluation of net benefit across multiple thresholds could be useful when optimising or comparing models.

In this paper, we show why, in order to consider the previous formulation of the area under the net benefit curve valid, an assumption of equal value of true positives across the population is needed. We introduce a new metric, called the continuous net benefit, derived from the conditional utility formula, and discuss its use, connecting it to performance metrics like the likelihood function and Brier score. The continuous net benefit requires an appropriately chosen weighting function across thresholds to be specified. We discuss the potential use of continuous net benefit to inform researchers of clinical utility during model validation, including examples of its application.

## Methods

### Introduction to the net benefit

Before introducing the continuous net benefit, we provide a brief walkthrough of the derivation of the net benefit as introduced in Vickers & Elkin (2006) in order to better position our proposed extension [4]. Here, the focus is not on determining whether a particular treatment is beneficial, but instead on determining the net benefit of a particular model when used to decide whether patients should receive treatment or not.

We explore this problem through an example in cardiovascular prognosis. Consider a population with a binary outcome of interest $\:Y$ which indicates whether an individual will have a cardiovascular event in the next ten years. A model is developed so that the risk of the outcome is estimated for each individual. Those with model scores above a threshold probability $\:t$ are prescribed statins, and the rest are not. The performance of that policy is summarised by its per capita true positives $\:TP\left(t\right)$ (number of people given statins who would have had a cardiovascular event if they had not been prescribed statins, divided by population size), false positives $\:FP\left(t\right)$ (people treated which would not have had the event if they had not been prescribed statins), false negatives $\:FN\left(t\right)$ (people who were not treated and who would then have the event) and true negatives $\:TN\left(t\right)$ (people who were not treated and would then not have the event). We define utility as a numerical measure of clinical benefit or harm. Its unit may vary depending on context, for example, quality-adjusted life years, probability of a favourable outcome, or patient-reported satisfaction. If $\:a$, $\:b$, $\:c$, and $\:d$ represent the utility for a patient of getting a true positive, false positive, false negative, and true negative result, respectively, the total utility, conditional on the population, of the model is defined as:

1 $$\:U\left(t\right)=aTP\left(t\right)+bFP\left(t\right)+cFN\left(t\right)+dTN\left(t\right)$$

The net benefit $\:NB$ is a simplified version of the utility so that if one model has a higher $\:NB$ than another, it also has a higher $\:U$. To derive the net benefit, the best choice of threshold, or clinically optimal threshold $\:{t}^{*}$ is determined by $\:\frac{1-{t}^{*}}{{t}^{*}}=\frac{a-c}{d-b}$, so that knowing the value of the utilities determines $\:{t}^{*}$, and thus knowing $\:{t}^{*}$ is informative of the utility values (without fully specifying them, as $\:{t}^{*}$ is a ratio of the four utilities) [4]. Rescaling the benefit of giving a patient statins when they would have had a cardiovascular event (i.e., $\:a-c$) to be 1, the net benefit becomes:

2 $$\:NB\left({t}^{*}\right)=TP\left({t}^{*}\right)-\frac{{t}^{*}}{1-{t}^{*}}FP\left({t}^{*}\right)$$

The optimal threshold $\:{t}^{*}$ can be determined from initial choices of the utilities $\:a$ to $\:d$, or more commonly through clinical knowledge and practice. If the optimal threshold $\:{t}^{*}$ is not known or varies across the population (as the utilities $\:a$ to $\:d$ might not be equal for everyone), the net benefit can be plotted across a reasonable range of values for $\:{t}^{*}$. This plot is known as the decision curve.

### Limitations of previous area under the net benefit curve metrics

A previous motivation for calculating the area under the net benefit curve has been the idea that individuals, due to different underlying utilities ($\:{a}^{*}$, $\:{b}^{*}$, $\:{c}^{*}$ and $\:{d}^{*}$), have different optimal thresholds $\:{t}^{*}$. For example, there will always be differences between individual patients in the extent to which they benefit from treatment or get harmed by side-effects. Models therefore need to be beneficial across a range of thresholds. One approach to account for this distribution of optimal thresholds $\:p\left({t}^{*}\right)$ is to check the decision curve throughout a previously determined range of reasonable thresholds (i.e., the range of $\:{t}^{*}$ for which we think $\:p\left({t}^{*}\right)$ is non-negligible). If the net benefit of a model is better than another policy across the entire range, the model is considered more beneficial [5]. This assumes that the threshold is a random variable $\:{T}^{*}$ independent of the performance of the model at that threshold, so that the overall estimate of the performance in terms of $\:TP\left({t}^{*}\right)$ and $\:FP\left({t}^{*}\right)$ is not biased compared to the subgroup-specific performance in those individuals for which the threshold is $\:{t}^{*}$.

The area under the net benefit curve has been proposed as a measure of the clinical benefit of a model in a population with a distribution of optimal thresholds $\:{T}^{*}$ by calculating its expected net benefit [11, 12]. It uses an estimated (or assumed) distribution of the optimal threshold $\:p\left({t}^{*}\right)$ to calculate the expected net benefit in a population:

3 $$\:AUNB={\int\:}_{0}^{1}p\left({t}^{*}\right)\left(TP\left({t}^{*}\right)-\frac{{t}^{*}}{1-{t}^{*}}FP\left({t}^{*}\right)\right)d{t}^{*}$$

Here we highlight an underlying assumption of this equation (but not of decision-curve analysis as a whole) which, to our knowledge, has not been previously discussed in the literature. Because the net benefit is in units of true positives, as mentioned in the previous section, giving $\:{a}^{*}-{c}^{*}=1$, then using the integral [3] to yield an expectation of the utility is only valid when assuming that true positives are equally beneficial for all patients. The difference in optimal thresholds across the population is thus entirely dictated by variations in the false positive harm ($\:{b}^{*}-{d}^{*}$) in each individual. This is unlikely to be true, as expected efficacy of treatment may drive differences in personal threshold.

While this is not an issue when examining the decision curve, as it is meant to be compared at every threshold (it is read ‘vertically’), it becomes an issue when the net benefit is integrated across a range (read ‘horizontally’). Let’s for example consider an alternative assumption, where the harm of a false positive $\:{b}^{*}-{d}^{*}$ is constant and equal to 1, so that $\:N{B}_{alt}=\frac{1-{t}^{*}}{{t}^{*}}TP\left({t}^{*}\right)-FP\left({t}^{*}\right)$, and the area under the net benefit is:

4 $$\:AUN{B}_{alt}={\int\:}_{0}^{1}p\left({t}^{*}\right)\left(\frac{1-{t}^{*}}{{t}^{*}}TP\left({t}^{*}\right)-FP\left({t}^{*}\right)\right)d{t}^{*}$$

For any single value of threshold $\:{t}^{*}$, $\:NB$ and $\:N{B}_{alt}$ are equivalent, in that if a model has higher net benefit than another in $\:NB$, it also has higher net benefit in $\:N{B}_{alt}$. This is however not true in terms of the integral as one model might have a higher $\:AUNB$ and lower $\:AUN{B}_{alt}$ than another (we show a proof for this in Additional file 1, Supplementary 1.4).

In general, knowing the distribution of optimal thresholds $\:p\left({t}^{*}\right)$ in a population of interest is not sufficient to calculate an expected or total benefit of a model in this population without additional knowledge or assumptions. To understand why this is the case, imagine a population of two groups $\:{G}_{1}$ and $\:{G}_{2}$ where each makes up half of the population. A prediction model is used to inform whether a preventative drug should be given in both groups, and this model has equal calibration and discrimination in $\:{G}_{1}$ and $\:{G}_{2}$. Consider that in group $\:{G}_{1}$, there is no real value in treatment nor harm in over-treating so that $\:{a}_{{G}_{1}}^{*}-{c}_{{G}_{1}}^{*}$ and $\:{b}_{{G}_{1}}^{*}-{d}_{{G}_{1}}^{*}$ are small, but balanced so that $\:{t}_{{G}_{1}}^{*}=\frac{{d}_{{G}_{1}}^{*}-{b}_{{G}_{1}}^{*}}{{d}_{{G}_{1}}^{*}-{b}_{{G}_{1}}^{*}+{a}_{{G}_{1}}^{*}-{c}_{{G}_{1}}^{*}}=10\%$. On the other hand, group $\:{G}_{2}$ is considered frail, and the treatment decision is ‘life-or-death’ for them, with large benefit in prevention, but serious harm in being given the drug when not needed, so $\:{a}_{{G}_{2}}^{*}-{c}_{{G}_{2}}^{*}$ and $\:{b}_{{G}_{2}}^{*}-{d}_{{G}_{2}}^{*}$ are large, but balanced so that $\:{t}_{{G}_{2}}^{*}=11\%$. A useful measure of the benefit of the model is not one that averages over the benefit of the model at $\:10\%$ and $\:11\%$ with equal weighting. In reality, decisions in group $\:{G}_{1}$ are inconsequential, and decisions in group $\:{G}_{2}$ are very important. It is thus important not only to know the distribution $\:p\left({t}^{*}\right)$ across the population, but also to have more information of the relative scale of the utilities $\:{a}^{*}-{c}^{*}$ and $\:{b}^{*}-{d}^{*}$, in order to know how useful a model is for a population with varying thresholds. This is not something you need to consider if you can separately optimise the choice of model in both groups but is essential if you are using a single model for both groups.

### Combining the net benefit of two decisions

We briefly focus on the related issue of combining the net benefit of multiple decisions. For example, we would now like to assess the net benefit of a cardiovascular model when both informing the prescription of statins and the enrolment of patients into a lifestyle intervention programme. It is often not necessary to combine the benefit of two interventions, as their benefit can be easily considered separately, and different models can be used for each decision [13]. However, considering how to do this is a useful intermediary step for later reasoning.

We assume that both decisions to intervene depend on the risk of the same outcome. The two interventions have different $\:{t}_{1}^{*}$ and $\:{t}_{2}^{*}$, different $\:{a}_{1}$, $\:{b}_{1}$, $\:{c}_{1}$, $\:{d}_{1}$ and $\:{a}_{2}$, $\:{b}_{2}$, $\:{c}_{2}$ and $\:{d}_{2}$, different $\:{U}_{1}$ and $\:{U}_{2}$, and net benefit values of $\:N{B}_{1}=NB\left({t}_{1}^{*}\right)$ and $\:N{B}_{2}=NB\left({t}_{2}^{*}\right)$. We assume that the effects of the two interventions do not interact, or that at least the utilities of the second intervention are in terms of the added benefit and harm with respect to the first, so that the conditional utility of both model-informed decisions is:

5 $$\begin{aligned}U_{1+2}&=U_1+U_2\\&=\:{a}_{1}TP\left({t}_{1}^{*}\right)+{b}_{1}FP\left({t}_{1}^{*}\right)+{c}_{1}FN\left({t}_{1}^{*}\right)+{d}_{1}TN\left({t}_{1}^{*}\right)\\& + a_2 TP(t_2^* )+b_2 FP(t_2^* )+c_2 FN(t_2^* )+d_2 TN(t_2^* )\end{aligned}$$

 

The goal is to find an expression of the net benefit $\:N{B}_{1+2}$ (i.e., the benefit of a model across the two interventions) so that, if and only if a model has a higher $\:{U}_{1+2}$ than another in a population, it also has a higher $\:{NB}_{1+2}$.

The first intuition for calculating the total net benefit $\:N{B}_{1+2}$ (the difference in benefit between using a model to inform which patients should get which treatments and never giving either treatment) is to choose $\:N{B}_{1+2}=N{B}_{1}+N{B}_{2}$. However, this is problematic, as $\:N{B}_{1}$ has as unit the benefit of treating a true positive with statins (so $\:{a}_{1}-{c}_{1}=1)$, and $\:N{B}_{2}$ has as unit the benefit of enrolling a true positive in a lifestyle intervention programme ($\:{a}_{2}-{c}_{2}=1$). The treatment effect of these two interventions is not equal ($\:{a}_{1}-{c}_{1}\ne\:{a}_{2}-{c}_{2}$). Instead, the ratio between the value of both interventions, $\:\frac{{a}_{2}-{c}_{2}}{{a}_{1}-{c}_{1}}$, needs to be estimated in order to calculate the total net benefit as:

6 $$\:N{B}_{1+2}=N{B}_{1}+\frac{{a}_{2}-{c}_{2}}{{a}_{1}-{c}_{1}}N{B}_{2}$$

In the cardiovascular example, if we estimate that enrolment in a lifestyle intervention programme is half as effective in reducing cardiovascular events compared to treating a patient with statins, an appropriate sum of the two net benefits which ranks models in the same order as $\:{U}_{1+2}$, would be $\:N{B}_{1+2}=N{B}_{1}+\frac{1}{2}N{B}_{2}$, up to a rescaling and offset factor.

### The continuous net benefit

In reality, cardiovascular prognostic models like QRISK are used for a very wide range of potential treatment strategies and monitoring plans that are generally impossible to enumerate in advance. We model this situation as a continuum of interventions, each with a particular optimal threshold $\:{t}^{{\prime\:}}$, so that, at each threshold $\:{t}^{{\prime\:}}$, the corresponding treatments carry an added true positive utility of $\:a{\prime\:}$ and false positive utility of $\:b{\prime\:}$. The total utility across all potential treatments for the patient is thus the integral of the conditional utility function:

7 $$\:{U}_{cont}={\int\:}_{0}^{1}a{\prime\:}TP\left({t}^{{\prime\:}}\right)+b{\prime\:}FP\left({t}^{{\prime\:}}\right)+cFN\left({t}^{{\prime\:}}\right)+dTN\left({t}^{{\prime\:}}\right)d{t}^{{\prime\:}}$$

Where $\:c$ and $\:d$ are constants, as they relate to the absence of any treatment. Writing $\:FN\left({t}^{{\prime\:}}\right)=\pi\:-TP\left({t}^{{\prime\:}}\right)$ and $\:TN\left({t}^{{\prime\:}}\right)=1-\pi\:-FP\left({t}^{{\prime\:}}\right)$, where $\:\pi\:=P(Y=1)$ is the incidence of the outcome in the population, we get:

8 $$\begin{aligned} \:\:{U}_{cont}=&\:d+\left(c-d\right)\pi\:+{\int\:}_{0}^{1}\left(a{\prime\:}-c\right)TP\left({t}^{{\prime\:}}\right)\\&+\left({b}^{{\prime\:}}-d\right)FP\left({t}^{{\prime\:}}\right)d{t}^{{\prime\:}} \end{aligned}$$

The term $\:d+\left(c-d\right)\pi\:$ is equal for all policies or models, and can thus be ignored when comparing models. For any threshold $\:{t}^{{\prime\:}}$, we still have $\:\frac{1-{t}^{{\prime\:}}}{{t}^{{\prime\:}}}=\frac{{a}^{{\prime\:}}-c}{d-{b}^{{\prime\:}}}$, and thus:

9 $$\:N{B}_{cont}={\int\:}_{0}^{1}{\omega\:}_{cont}\left({t}^{{\prime\:}}\right)(\frac{TP\left({t}^{{\prime\:}}\right)}{{t}^{{\prime\:}}}-\frac{FP\left({t}^{{\prime\:}}\right)}{1-{t}^{{\prime\:}}})d{t}^{{\prime\:}}$$

**Fig. 1:**
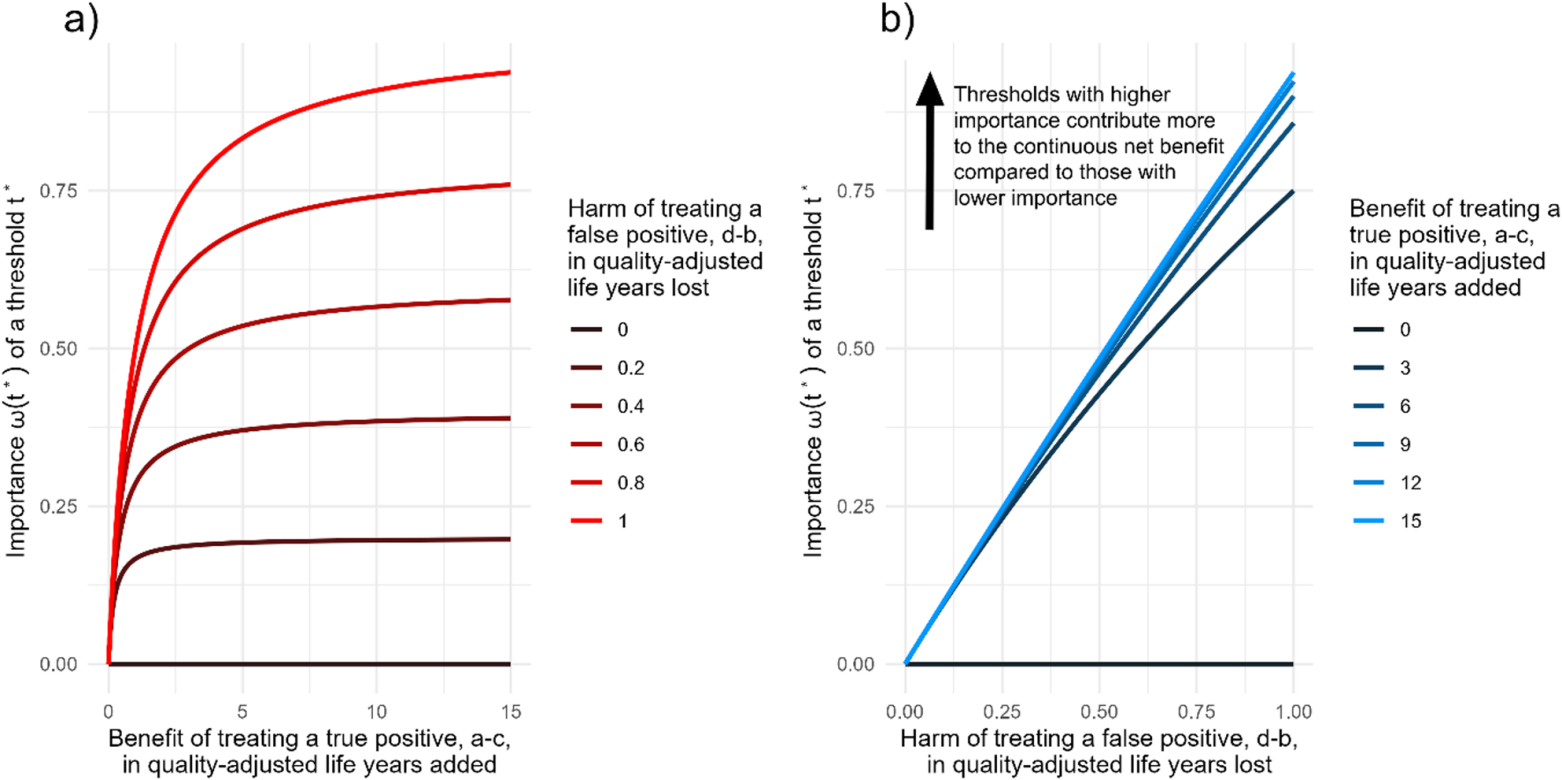
Change in the value of the importance of a threshold $\:{t}^{*}=\frac{1}{1+\frac{a-c}{d-b}}$ as part of the weighting function $\:\omega\:\left({t}^{*}\right)=\frac{1}{\frac{1}{a-c}+\frac{1}{d-b}}$ over different values of $\:a-c$ and $\:d-b$, where the two utility differences are in units of quality-adjusted life years added $\:(a-c)$ or lost $\:(d-b)$ when a patient is flagged positive. These plots show how, in this example, the value of $\:\omega\:$ changes more as $\:d-b$, varies, as it is the smallest out of the two utility differences

We call this metric the continuous net benefit. The sum $\:\frac{TP\left({t}^{{\prime\:}}\right)}{{t}^{{\prime\:}}}-\frac{FP\left({t}^{{\prime\:}}\right)}{1-{t}^{{\prime\:}}}$ is equal, up to a constant, to the sum of the net benefit in the treated and that in the untreated for an intervention with optimal threshold $\:{t}^{{\prime\:}}$ [14]. The weighting function $\:{\omega\:}_{cont}\left({t}^{{\prime\:}}\right)=\frac{1}{\frac{1}{{a}^{{\prime\:}}-c}+\frac{1}{d-{b}^{{\prime\:}}}}$ is the harmonic mean between the utility of identifying a true positive $\:{a}^{{\prime\:}}-c$, and avoiding a false positive $\:d-{b}^{{\prime\:}}$. We call the value of this weighting function for a particular threshold its ‘importance’, as it, in practice, determines how much the benefit (and thus performance) of a model at a particular threshold should be weighed against other thresholds. If a normalisation constant is chosen so that $\:{\int\:}_{0}^{1}\frac{{\omega\:}_{cont}\left({t}^{{\prime\:}}\right)}{{t}^{{\prime\:}}}d\:{t}^{{\prime\:}}=1$, the unit of the continuous net benefit is still true positives (though it will be combined true positives, i.e., the benefit that one patient with $\:Y=1$ gets when managed as a high-risk patient for all decisions). The continuous net benefit represents the net utility gain, relative to a baseline ‘treat-none’ strategy, when the model informs all treatments under consideration. Its units are true positives. A continuous net benefit of one indicates that, compared with classifying everyone as low risk (and therefore treating no one), the model provides a population-level benefit equivalent to correctly identifying and fully treating one additional true positive patient. The continuous net benefit can also be calculated for non-model-based policies, such as treating all patients as low risk (treat-none) or as high risk (treat-all). For the treat-none policy, the continuous net benefit is always zero, as is the case for the standard net benefit, as it represents the reference strategy against which net gain or loss is measured. Comparing any model with these two baseline policies is recommended to determine whether it provides benefit or causes harm relative to much simpler alternatives. Generally, this weighting function is high only when both the utilities $\:a{\prime\:}-c$ and $\:d-b{\prime\:}$ are high, and is low if either of the two is low. To visualise this, consider possible treatments for cardiovascular disease, and the quality-adjusted life years (QALYs) added or lost due to true and false positives. We might expect the benefit of true positives to range between 5 and 15 QALYs compared to not intervening, and the harms of false positives to be lower, between 0.1 and 1 QALYs lost compared to not intervening, as commonly used preventive interventions in cardiovascular care do not usually carry serious side-effects. As seen in Fig. 1, in this case, the importance of a threshold is mostly determined by the severity of the harm of false positives, as it is the smaller effect in the harmonic mean, meaning that interventions with worse potential false positive harm should be weighted more than those with relatively low harm.

Some metrics commonly used to assess the performance of prediction models can be related to the continuous net benefit through specific choices of weighting function. For example, consider the choice of a uniform function $\:{\omega\:}_{cont}\left({t}^{{\prime\:}}\right)=1$. The chosen ‘importance’ of all thresholds is thus equal. In this case, $\:N{B}_{cont}={\int\:}_{0}^{1}\frac{TP\left({t}^{{\prime\:}}\right)}{{t}^{{\prime\:}}}-\frac{FP\left({t}^{{\prime\:}}\right)}{1-{t}^{{\prime\:}}}d{t}^{{\prime\:}}$, and the difference between the continuous net benefit of two models $\:{M}_{1}$ and $\:{M}_{2}$ is equivalent to the difference between their likelihoods, with:

10 $$\begin{aligned} \:{\omega\:}_{cont}\left({t}^{{\prime\:}}\right)&=1\to\:N{B}_{cont}\left({M}_{1}\right)-N{B}_{cont}\left({M}_{2}\right)\\&=\mathcal{L}\left({M}_{1}\right)-\mathcal{L}\left({M}_{2}\right) \end{aligned}$$

Here, $\:\mathcal{L}$ is the likelihood function of the models given the observed data. The derivation of this result is shown in Additional file 1, Supplementary 1.1. Models are often fitted to maximise the likelihood function, which could intuitively be interpreted as choosing the best model when considering each threshold equally important. On the other hand, if a weighting function is chosen to be a symmetric parabola $\:{\omega\:}_{cont}\left({t}^{{\prime\:}}\right)={t}^{{\prime\:}}(1-{t}^{{\prime\:}})$ so that $\:N{B}_{cont}={\int\:}_{0}^{1}{t}^{{\prime\:}}(1-{t}^{{\prime\:}})(\frac{TP\left({t}^{{\prime\:}}\right)}{{t}^{{\prime\:}}}-\frac{FP\left({t}^{{\prime\:}}\right)}{1-{t}^{{\prime\:}}})d{t}^{{\prime\:}}$, the difference between the continuous net benefit of two models is equivalent to the negative Brier score difference between them, with:

11 $$\begin{aligned} \:{\omega\:}_{cont}\left({t}^{{\prime\:}}\right)&={t}^{{\prime\:}}\left(1-{t}^{{\prime\:}}\right)\to\:N{B}_{cont}\left({M}_{1}\right)-N{B}_{cont}\left({M}_{2}\right)\\&=\frac{1}{2}\left(-Brier\left({M}_{1}\right)+Brier\left({M}_{2}\right)\right) \end{aligned}$$

The derivation of this result is shown in Additional file 1, Supplementary 1.2. The Brier score has been found to be a suboptimal metric to represent clinical benefit [15]. This follows from its associated parabolic weighting function: in most clinical prediction model applications, the most important thresholds rarely lie near 0.5, as true positive benefit typically outweighs false positive harm, leading to weighting functions with higher values at lower thresholds. Finally, the binary net benefit of a model can be recovered by using a point mass as the weighting function $\:{\omega\:}_{cont}\left({t}^{{\prime\:}}\right)=\delta\:({t}^{{\prime\:}}-{t}^{*})\:$, giving $\:N{B}_{cont}={\int\:}_{0}^{1}\delta\:\left({t}^{{\prime\:}}-{t}^{*}\right)\left(\frac{TP\left({t}^{{\prime\:}}\right)}{{t}^{{\prime\:}}}-\frac{FP\left({t}^{{\prime\:}}\right)}{1-{t}^{{\prime\:}}}\right)d{t}^{{\prime\:}}=\frac{TP\left({t}^{*}\right)}{{t}^{*}}-\frac{FP\left({t}^{*}\right)}{1-{t}^{*}}$.

To choose a weighting function in practice, we recommend first defining a plausible range of thresholds where clinical decisions are made. Researchers can then divide this range according to intervention types and qualitatively compare expected true positive benefits and false positive harms. Regions where both benefits and harms are large should be assigned greater importance than regions where both are small. Within subranges corresponding to similar interventions, the shape of the weighting function should reflect how these benefits and harms change across thresholds. If higher thresholds correspond to greater false positive harm, the weighting function should increase with the threshold. Conversely, if higher thresholds reflect diminishing returns in true positive benefit, it should decrease. When both vary, the weighting function will be mainly driven by the smaller of the two effects, the benefit $\:{a}^{{\prime\:}}-c$ and the harm $\:d-{b}^{{\prime\:}}$, as shown in Fig. 1. Researchers can elicit this information from clinicians by asking about the range of risk scores at which decisions are made and the main factors (treatment benefit or risk) influencing threshold changes. These considerations allow researchers to sketch a plausible weighting function, which can then be approximated by a smooth distribution to compute the continuous net benefit. Alternatively, expected changes in the utilities $\:{a}^{{\prime\:}}$ and $\:{b}^{{\prime\:}}$ across the range can be simulated, and using the formula $\:{\omega\:}_{cont}\left({t}^{{\prime\:}}\right)=\frac{1}{\frac{1}{{a}^{{\prime\:}}-c}+\frac{1}{d-{b}^{{\prime\:}}}}$, used to derive the corresponding weighting function directly.

### Considering single interventions with varying thresholds using the continuous net benefit

We revisit the motivation of previous literature of calculating an expected net benefit in a population with a distribution of utilities $\:{a}^{*}$, $\:{b}^{*}$, $\:{c}^{*}$ and $\:{d}^{*}$, and a corresponding distribution of optimal threshold $\:\frac{1-{t}^{*}}{{t}^{*}}=\frac{{a}^{*}-{c}^{*}}{{d}^{*}-{b}^{*}}$. In that case, the expected utility of the population is:

12 $$\begin{aligned} \:{U}_{E}=&\:{\int\:}_{0}^{1}p\left({t}^{*}\right)\left({a}^{*}TP\left({t}^{*}\right)+{b}^{*}FP\left({t}^{*}\right)+{c}^{*}FN\left({t}^{*}\right) \right. \\& \left. +\:{d}^{*}TN\left({t}^{*}\right)\right)d{t}^{*} \end{aligned}$$

Following a similar derivation to that from Eq. (7) to (9), detailed in Additional file 1, Supplementary 1.3, the expected net benefit across the population can be obtained as:

13 $$\:N{B}_{E}={\int\:}_{0}^{1}{\omega\:}_{E}\left({t}^{*}\right)(\frac{TP\left({t}^{*}\right)}{{t}^{*}}-\frac{FP\left({t}^{*}\right)}{1-{t}^{*}})d{t}^{*}$$

Where the weighting function $\:{\omega\:}_{E}\left({t}^{*}\right)=\frac{p\left({t}^{*}\right)}{\frac{1}{{a}^{*}-{c}^{*}}+\frac{1}{{d}^{*}-{b}^{*}}}$ includes both the distribution of the thresholds across the population and the utilities for each of these thresholds. In this case, defining a weighting function follows a similar process to that described in the previous section, where now the focus shifts to changes in utility across patients, not treatments. A second step is now to estimate or assume a threshold distribution $\:p\left({t}^{*}\right)$ and multiply it by the weighting function to obtain $\:{\omega\:}_{E}\left({t}^{*}\right)$. If the weighting function is normalised so that $\:{\int\:}_{0}^{1}\frac{{\omega\:}_{E}\left({t}^{*}\right)}{{t}^{*}}d\:{t}^{*}=1$, the unit of the net benefit will be true positives across everyone in the population. As each patient has distinct utilities ($\:{a}^{*}$ to $\:{d}^{*}$) and corresponding thresholds ($\:{t}^{*}$), the benefit of treating a true positive is different across individuals. The continuous net benefit unit thus represents the *average* utility gain from treating a true positive. A continuous net benefit of one true positive indicates that, compared with the treat-none strategy, the model yields an average utility gain equivalent to correctly identifying and treating one true positive patient as high risk. R code showcasing how to calculate the continuous net benefit is included in Additional file 2.

## Results

### Example 1: developing a cardiovascular risk prognostic model for multiple interventions

We showcase the use of the continuous net benefit through an example. We develop and validate four models and policies (Full Model, Dichotomised Model, Small Model, and Marker-based Policy, detailed in Additional File 3, Supplementary 3.4) to predict the risk that an individual will have a cardiovascular disease event in the next 10 years, using data from the Framingham Heart Study [16]. 

We evaluate the models for two separate sets of decisions: (1) the prescription of statins, evaluated at the optimal threshold of $\:10\%$ used in UK practice [7], and (2) overall patient management and lifestyle recommendations. The first is evaluated by placing a point mass (i.e., calculating the standard net benefit) at the 10% threshold, while the second is evaluated using the continuous net benefit, choosing as weighting function $\:{\omega\:}_{cont}$ a half-Gaussian, corresponding to a full-Gaussian with mean of $\:10\%$ and standard deviation of $\:2\%$ with only non-zero values below $\:10\%$. This weighting function was chosen after discussion with a clinician, after which we determined that usually clinicians will attempt to reduce the risk of a patient through lifestyle interventions and more frequent monitoring before their risk reaches $\:10\%$. We believe that these interventions are especially ‘aggressive’ as the patient gets closer to the threshold of 10%, and carry higher risks, like those of exercise-related injuries or disengagement from healthcare. To capture this, we specified an increasing weighting function over the relevant range (5% to 10%), giving greater importance to higher thresholds where potential harms are larger, without meaningful changes in true positive benefit. The standard deviation of 2% was chosen to reflect that thresholds below 5% are clinically negligible. To assess the sensitivity of the metric to misspecified weighting functions, we also evaluated a uniformly weighted area under the standard net benefit, as defined in expression [2], across thresholds from 5% to 10%. Further details on the development and validation of the model are included in Additional file 3, and full code is included in Additional file 4. Confidence intervals (95%CI) and optimism corrections for the continuous net benefit are calculated through bootstrapping [17]. Although, from a ‘pure’ decision theory standpoint, it is not necessary to report confidence intervals for decision curves [18], we still report them for the continuous net benefit to show the expected variance of the proposed metric. Here, the confidence interval should be interpreted in the same way as it is interpreted for the standard net benefit, as reflecting only the variability due to sample selection. A 95% confidence interval indicates the range that would contain the population mean continuous net benefit, given the chosen weighting function, in 95% of repeated samples. Importantly, these intervals do not capture uncertainty about the optimal threshold or about whether individual patients will benefit from the model.

**Fig. 2:**
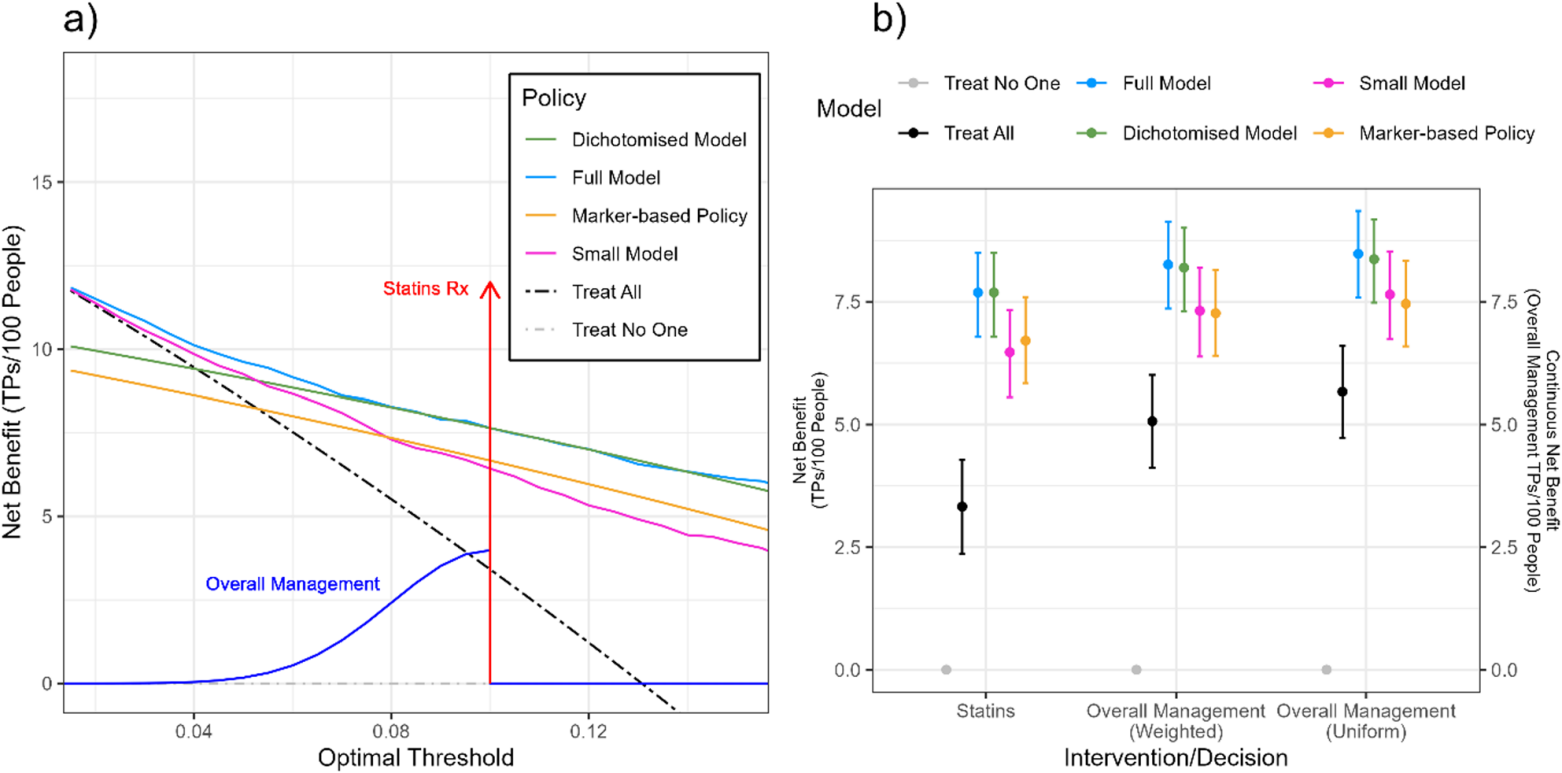
Reported benefit of the cardiovascular model development example when considering multiple treatments. **a** Decision curve of the developed logistic regression model using all (Full Model), a dichotomised version of the same logistic regression, where patients are only shown as low or high risk (Dichotomised Model), a logistic regression with a limited set of the available predictors (Small Model), or a policy based on whether a patient has one of four markers associated with high risk (Marker-based Policy). We compare these models to the policies of treating all patients (Treat All) and treating no patients (Treat No One). The weighting function to calculate the continuous net benefit function is shown, separated into the components related to the prescription of statins (red) and the recommendation of lifestyle changes (blue). **b** Continuous net benefit estimates of all policies for the decisions of prescribing statins and overall patient management, as well as a uniformly weighted area under the net benefit curve between the thresholds of 5% and 10%. The unit of the statins component is the benefit of treating a true positive patient with statins per 100 people, and the unit of the two overall management components is the benefit of managing a true positive patient as high risk per 100 people

The decision curves of the models, as well as the weighting function chosen, are plotted in Fig. 2a, and the net benefit of both sets of interventions is shown in Fig. 2b. For overall patient management (excluding statins), when the weighting function was appropriately chosen, the continuous net benefit of the Full Model was highest, 8.3 (95%CI: 7.4–9.1) true positives per 100 people, followed by the Dichotomised Model (8.2, 7.3–9.0), the Small Model (7.3, 6.4–8.2) and finally the Marker-based Policy (7.3, 6.4–8.1). The results of the uniformly weighted continuous net benefit produced slightly different absolute values of the net benefit, but preserved the previous ranking, with the Full Model (8.5, 7.6–9.4), the Dichotomised Model (8.4, 7.5–9.2), the Small Model (7.7, 6.7–8.5), and the Marker-based Policy (7.5, 6.6–8.3) ranking from most to least beneficial. When looking at statins prescribing (i.e., the net benefit at the 10% threshold), the Full Model and Dichotomised model naturally had the same net benefit (7.7, 6.8–8.5), followed by the Marker-based Policy (6.7, 5.8–7.6) and finally the Small Model (6.5, 5.6–7.3).

### Example 2: Developing a cardiovascular risk prognostic model for one intervention with a distribution of thresholds

**Fig. 3:**
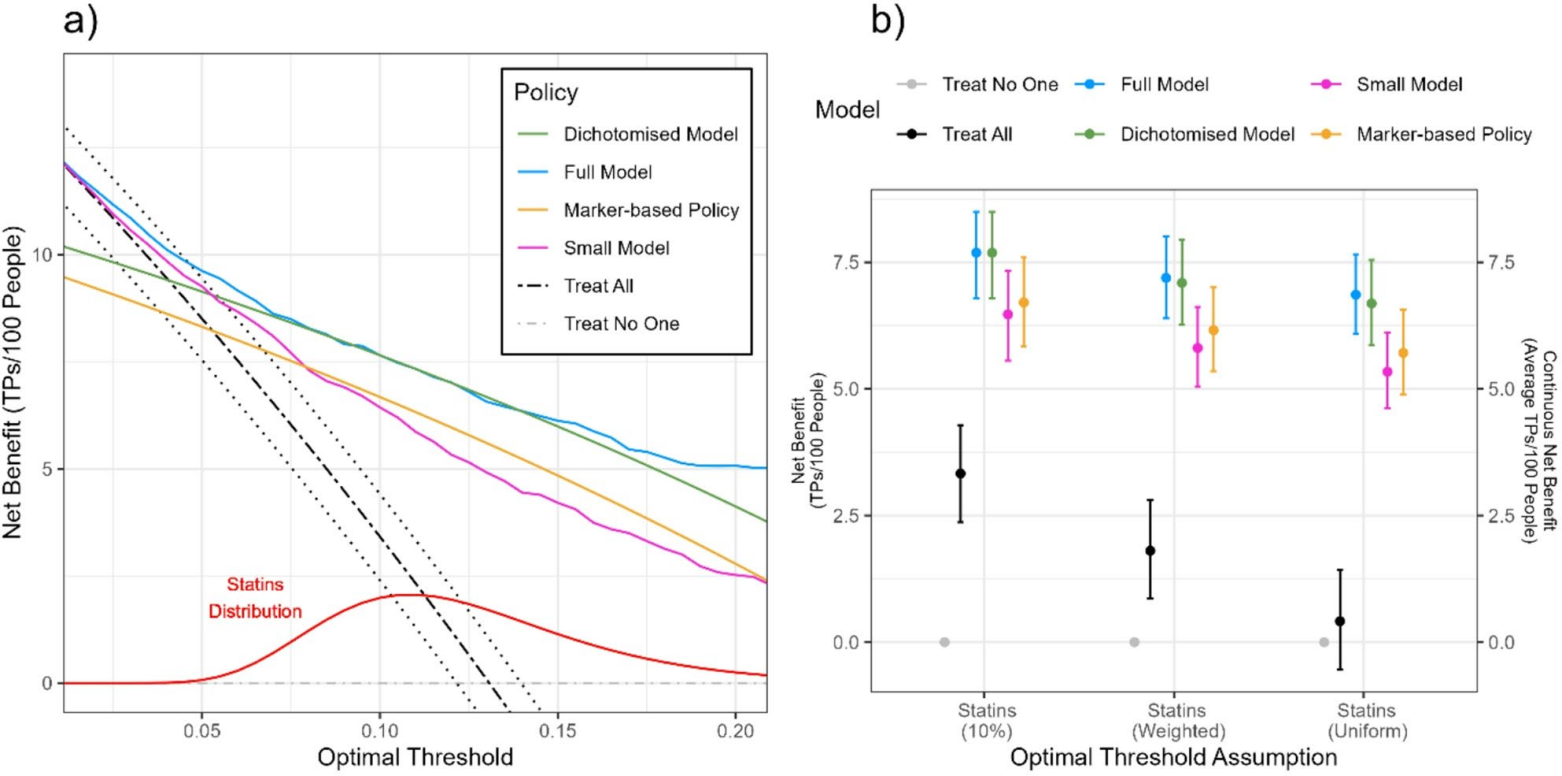
Reported benefit of the cardiovascular model development example when considering a single treatment with a distribution of optimal thresholds across the population. **a** Decision curve of the developed logistic regression model using all (Full Model), a dichotomised version of the same logistic regression (Dichotomised Model), a logistic regression with a limited set of the available predictors (Small Model), or a policy based on whether a patient has one of four markers associated with high risk (Marker-based Policy). We compare these models to the policies of treating all patients (Treat All) and treating no patients (Treat No One). The assumed distribution of optimal thresholds across the population is shown in red. **b** Continuous net benefit estimates of all policies for the decision of prescribing statins, with different assumptions of everyone having an optimal threshold of 10% (left), individuals having optimal thresholds taken from the distribution shown in (a) (middle), and using a uniformly weighted area under the net benefit curve between the thresholds of 5% and 20% (right). The unit is the average benefit of treating a true positive patient with statins per 100 people

In this second example, we instead consider that not all individuals will be prescribed statins when they are over the risk of $\:10\%$. Usually, the range of thresholds at which cardiovascular models are evaluated in decision curve analysis goes between 0% and 20% [6], although higher thresholds might be considered [19]. We model the distribution of optimal thresholds $\:p\left({t}^{*}\right)$ to be a log normal distribution with mean of 0.12 and logarithm standard deviation of 0.3, plotted in Fig. 3a. We think that the main variation of thresholds is due to changes in the relative risk reduction achieved by statins, and we thus assume that the harms due to false positives are constant ($\:d-b=1$), so that we use Eq. (4) to calculate an expected net benefit across the population. If we wanted to consider variation in false positive harm in our analysis (as in reality, some patients value more negatively false positive harm from preventative treatment), Eq. (13) would need to be used. We have also evaluated a uniformly weighted area under the standard net benefit between thresholds of 5% and 20%.

The continuous net benefit, shown in Fig. 3b, in units of average true positives per 100 people, is 7.2 (6.4–8.0) for the Full Model, 7.1 (6.3–8.0) for the Dichotomised Model, 5.8 (5.0–6.6) for the Small Model, and 6.2 (5.3–7.0) for the Marker-based Policy. Generally, the continuous net benefit was in agreement with the net benefit at the threshold of 10% and the uniformly weighted area under the net benefit.

## Discussion

In this paper, we present an approach to assess the clinical utility of prognostic models in situations where the model is used for overall patient management, or situations where we believe that a single intervention’s optimal threshold varies across the population. Summarising the total net benefit as a single measure showed the overall usefulness of prediction models in a cardiovascular problem.

In both examples, the continuous net benefit produced results similar to those from the single-threshold net benefit and from a misspecified version using a uniform weighting function. This is in part due to the evaluated policies being ranked consistently across the entire relevant threshold range. Such agreement may not hold when the relevant threshold range is wider or when models differ more substantially. For example, models which rely on different predictor sets may perform differently when discriminating in high-risk or low-risk patients. Further work is needed to identify the conditions under which simpler approaches, such as using a single threshold or an imprecise weighting function, yield acceptable approximations. Even when differences between approaches are small, the continuous net benefit remains, in principle, the more appropriate method for summarising model value across multiple thresholds, as it provides absolute benefit estimates that more accurately represent overall population utility. It can therefore serve as a reference for determining when simplifications, such as single-threshold evaluation, are justified.

In both examples, the continuous net benefit produced results similar to those from the single-threshold net benefit and from a misspecified version using a uniform weighting function. This is in part due to the evaluated policies being ranked consistently across the entire relevant threshold range. Such agreement may not hold when the relevant threshold range is wider or when models differ more substantially. For example, models which rely on different predictor sets may perform differently when discriminating in high-risk or low-risk patients. Further work is needed to identify the conditions under which simpler approaches, such as using a single threshold or an imprecise weighting function, yield acceptable approximations. Even when differences between approaches are small, the continuous net benefit remains, in principle, the more appropriate method for summarising model value across multiple thresholds, as it provides absolute benefit estimates that more accurately represent overall population utility. It can therefore serve as a reference for determining when simplifications, such as single-threshold evaluation, are justified.

We recommend using the continuous net benefit when assessing model benefit across a range of thresholds is important, for example when considering the distribution of optimal thresholds across the population, or when multiple clinical decisions are considered simultaneously. We believe it especially adds value when a quantitative assessment of the overall benefit difference between models or policies is needed, and graphical assessments using decision curves are not enough. For instance, a difficult-to-implement complex model might have a higher decision curve than a simpler one, but without quantifying the magnitude of its added clinical utility, it is difficult to judge whether implementation is justified. Besides using it during the validation stage, the continuous net benefit could also be useful for researchers and methodologists during model development, when comparing modelling choices and when needing a metric to optimise [20], or when summarising the benefit of a model over multiple validation studies using meta-analysis [21]. 

Use of the continuous net benefit may not be warranted in situations where the graphical assessment of using decision curves is sufficient, such as when, for example, a particular model is vastly superior to all other policies across the entire range. Moreover, if a reasonable weighting function that reflects the clinical problem cannot be roughly estimated, the metric loses clinical meaning and practical interpretability. Importantly, we do not recommend to ever use the continuous net benefit as a substitute of decision curves, but rather as an addition to the framework, similarly to how calibration-in-the-large and the calibration slope are reported alongside calibration plots. While the continuous net benefit is limited in its ability to assess clinical utility by the assumptions discussed, we nonetheless consider that it can be a useful first step before carrying out more involved cost-benefit analysis further down the implementation of a clinical prediction model.

## Conclusions

An increasing number of clinical prediction models are being created, with few being implemented in practice [22, 23]. In part, this is due to the large number of available models, and to the challenges faced by policymakers when inferring whether models would be clinically useful and beneficial to patients. Our work introduces to clinical prediction model researchers a tool that can help decision makers better consider the use of models beyond a single systematic clinical decision, enabling them to assess the overall clinical value of an algorithm. Further work could investigate how assumptions around the independence of optimal thresholds and predictors can be relaxed with subgroup analysis or explore different approaches to choosing the weighting function.

## Supplementary Information

Additional file 1. Supplementary containing proofs of some statements made in the Methods section

Additional file 2. (.R): Code (in R) showcasing the use of continuous net benefit

Additional file 3. Supplementary containing additional information of the model development and validation presented in the Results section

Additional file 4. (.R): Full code (in R) of the model development and validation presented in the Results section

## Data Availability

The data used in the two examples is publicly available as part of the ‘riskCommunicator’ package in R. All code used for this work, which includes importing the data, is included as an additional file.

## Abbreviations

QALY: Quality-Adjusted Life Years
