# Supplementary material for: The continuous net benefit: assessing the clinical utility of prediction models when informing a continuum of decisions
Source: Diagn Progn Res. 2026 Feb 17;10:8. doi: 10.1186/s41512-026-00224-z (PMC12911006; doi:10.1186/s41512-026-00224-z)
Supplement: Supplementary file 1 — Additional file 1. Supplementary containing proofs of some statements made in the Methods section [file 41512_2026_224_MOESM1_ESM.docx]

*Supplementary 1.1: Proof that a uniform weighting function yields the likelihood function when calculating the continuous net benefit.*

We seek to prove the statement that, when choosing a uniform weighting function $\omega_{cont}\left( t^{'} \right)=1$, the difference in continuous net benefit between two models is equivalent to their difference in the likelihood function. In order to write the proof, we will write the true and false positives for a model $f:X\to Y$ on a dataset $\left\{ x_{i},y_{i} \right\}_{i=1,2\ldots N}$ as:

$$TP\left( t \right)= \sum_{i} \theta\left( f\left( x_{i} \right)-t \right)y_{i}$$

$$FP\left( t \right)= \sum_{i} \theta\left( f\left( x_{i} \right)-t \right)\left( 1-y_{i} \right)$$

Where $\theta$ is a step function equal to 1 if $f\left( x_{i} \right)-t>0$, and 0 if $f\left( x_{i} \right)-t\leq0$. We consider two models $f_{1},f_{2}:X\to(0,1)$ with true and false positives $TP_{1}(t)$, $TP_{2}(t)$, $FP_{1}(t)$ and $FP_{2}(t)$. Their difference in continuous net benefit is thus:

$$NB_{cont1}-NB_{cont2}=\int_{0}^{1} \frac{TP_{1}\left( t^{'} \right)}{t^{'}}-\frac{FP_{1}\left( t^{'} \right)}{1-t^{'}}dt^{'}- \int_{0}^{1} \frac{TP_{2}\left( t^{'} \right)}{t^{'}}-\frac{FP_{2}\left( t^{'} \right)}{1-t^{'}}dt^{'}$$

$$=\sum_{i} \int_{0}^{1} \frac{\theta\left( f_{1}\left( x_{i} \right)-t^{'} \right)y_{i}}{t^{'}}-\frac{\theta\left( f_{1}\left( x_{i} \right)-t^{'} \right)\left( 1-y_{i} \right)}{1-t^{'}}dt^{'}- \int_{0}^{1} \frac{\theta\left( f_{2}\left( x_{i} \right)-t^{'} \right)y_{i}}{t^{'}}-\frac{\theta\left( f_{2}\left( x_{i} \right)-t^{'} \right)\left( 1-y_{i} \right)}{1-t^{'}}dt^{'}$$

$$=\sum_{i} \int_{0}^{f_{1}\left( x_{i} \right)} \frac{y_{i}}{t^{'}}-\frac{1-y_{i}}{1-t^{'}}dt^{'}- \int_{0}^{f_{2}\left( x_{i} \right)} \frac{y_{i}}{t^{'}}-\frac{1-y_{i}}{1-t^{'}}dt^{'}$$

$$=\sum_{i} \int_{0}^{f_{1}\left( x_{i} \right)} \frac{y_{i}}{t^{'}}+\frac{y_{i}}{1-t^{'}}dt^{'}-\int_{0}^{f_{1}\left( x_{i} \right)} \frac{1}{1-t^{'}}dt^{'}- \int_{0}^{f_{2}\left( x_{i} \right)} \frac{y_{i}}{t^{'}}+\frac{y_{i}}{1-t^{'}}dt^{'}+\int_{0}^{f_{2}\left( x_{i} \right)} \frac{1}{1-t^{'}}dt^{'}$$

$$= \sum_{i} y_{i}(\log\left( t^{'} \right)-\log\left( 1-t^{'} \right)\left. \right|_{t^{'}=0}^{t^{'}=f_{1}\left( x_{i} \right)}+(\log\left( 1-t^{'} \right)\left. \right|_{t^{'}=0}^{t^{'}=f_{1}\left( x_{i} \right)} -y_{i}(\log\left( t^{'} \right)-\log\left( 1-t^{'} \right)\left. \right|_{t^{'}=0}^{t^{'}=f_{2}\left( x_{i} \right)}-(\log\left( 1-t^{'} \right)\left. \right|_{t^{'}=0}^{t^{'}=f_{2}\left( x_{i} \right)}$$

At this point, the left extremes of the evaluation of the integrals cancels out as it is equal in the two models. This cancellation can be seen if we instead take the integral between $\epsilon$ and 1, so that $\log\left( \epsilon\right)$ is well defined, and take the limit $\epsilon\to0$ after $log(\epsilon)$ is cancelled in the difference of net benefit between the two models.

$$NB_{cont1}-NB_{cont2}=\sum_{i} y_{i}\log\left( f_{1}\left( x_{i} \right) \right)-y_{i}\log\left( {1-f}_{1}\left( x_{i} \right) \right)+\log\left( {1-f}_{1}\left( x_{i} \right) \right)-y_{i}\log\left( f_{2}\left( x_{i} \right) \right)+y_{i}\log\left( {1-f}_{2}\left( x_{i} \right) \right)-\log\left( {1-f}_{2}\left( x_{i} \right) \right)$$

$$= \sum_{i} y_{i}\log\left( f_{1}\left( x_{i} \right) \right)+\left( 1-y_{i} \right)\log\left( 1-f_{1}\left( x_{i} \right) \right)-\sum_{i} y_{i}\log\left( f_{2}\left( x_{i} \right) \right)+\left( 1-y_{i} \right)\log\left( 1-f_{2}\left( x_{i} \right) \right)$$

$$\mathcal{=L}\left( f_{1},\left\{ x_{i},y_{i} \right\}_{i=1,2\ldots N} \right)\mathcal{-L}\left( f_{1},\left\{ x_{i},y_{i} \right\}_{i=1,2\ldots N} \right) \square$$

Where $\mathcal{L}$ is the likelihood function of the model for the observed data.

*Supplementary 1.2: Proof that a parabola weighting function yields the Brier score when calculating the continuous net benefit.*

The proof for this statement starts in the same way as that of Supplementary 1.1. We consider the weighting function $\omega_{cont}\left( t^{'} \right)=t^{'}(1-t^{'})$ to calculate the continuous net benefit of a model $f_{1}$.

$$NB_{cont1}=\int_{0}^{1} t^{'}\left( 1-t^{'} \right)\left( \frac{TP_{1}\left( t^{'} \right)}{t^{'}}-\frac{FP_{1}\left( t^{'} \right)}{1-t^{'}} \right)dt^{'}$$

$$=\sum_{i} \int_{0}^{1} \theta\left( f_{1}\left( x_{i} \right)-t^{'} \right)y_{i}\left( 1-t^{'} \right)-\theta\left( f_{1}\left( x_{i} \right)-t^{'} \right)\left( 1-y_{i} \right)t^{'}dt^{'}$$

$$= \sum_{i} \int_{0}^{f_{1}\left( x_{i} \right)} y_{i}-y_{i}t^{'}-t^{'}+y_{i}t^{'}dt^{'}$$

$$= \sum_{i} (y_{i}t^{'}-\frac{1}{2}\left( t^{'} \right)^{2}\left. \right|_{t^{'}=0}^{t^{'}=f_{1}\left( x_{i} \right)}$$

$$=-\frac{1}{2}\sum_{i} {-2y}_{i}f_{1}\left( x_{i} \right)+f_{1}\left( x_{i} \right)^{2}+y_{i}^{2}-y_{i}^{2}$$

$$=-\frac{1}{2}\sum_{i} \left( y_{i}-f_{1}\left( x_{i} \right) \right)^{2}+\frac{1}{2}\sum_{i} y_{i}^{2}$$

$$=-\frac{1}{2}Brier\left( f_{1},\left\{ x_{i},y_{i} \right\}_{i=1,2\ldots N} \right)+\frac{1}{2}\pi$$

Where $\pi$ is the prevalence of the outcome in the population. Thus, by comparing the continuous net benefits of two models $f_{1}$ and $f_{2}$ we obtain:

$$NB_{cont1}-NB_{cont2}=\frac{1}{2}\left( -Brier\left( f_{1},\left\{ x_{i},y_{i} \right\}_{i=1,2\ldots N} \right)+Brier\left( f_{2},\left\{ x_{i},y_{i} \right\}_{i=1,2\ldots N} \right) \right) \square$$

*Supplementary 1.3: Calculating the expected net benefit for a population with a distribution of utilities a, b, c and d.*

Assume that we have a population $\left\{ x_{i},y_{i} \right\}_{i=1,2\ldots N}\sim X,Y$ where each individual has a unique set of utilities $\left\{ a_{i},b_{i},c_{i},d_{i} \right\}_{i=1,2\ldots N}\sim A,B,C,D$. We assume that we do not know the individual values of $a_{i}$, $b_{i}$, $c_{i}$ and $d_{i}$ for each individual, nor the distribution of $A$, $B$, $C$ and $D$. Instead, we know the distribution of thresholds $T^{*}$ where $t^{*}\sim T^{*}$ is the optimal threshold for each individual, with $\frac{1-t^{*}}{t^{*}}=\frac{a-c}{d-b}$, but do not have individual values $t_{i}^{*}$. We consider a prediction model $f:X\to Y$ which gives each individual a score $\left\{ f\left( x_{i} \right) \right\}_{i=1,2\ldots N}$.

The conditional utility of the model for any arbitrary threshold $t$ is distributed across the population as:

$$U\left( f(X)=f(x),A=a,B=b,C=c,D=d \right)=aP\left( Y=1,f\left( x \right)>t | a,b,c,d \right)$$

$$+bP\left( Y=0,f\left( x \right)>t | a,b,c,d \right)+cP\left( Y=1,f\left( x \right)<t | a,b,c,d \right)$$

$$+dP(Y=0,f\left( x \right)<t|a,b,c,d)$$

We assume that the coefficients of the utility $A$, $B$, $C$ and $D$ are independent of the performance (true and false positives and negative rates) of the model. This assumption is needed as otherwise the overall proportion of true positives at a particular threshold would not be equal to the proportion of true positives in the subgroup for which that threshold is optimal. This assumption is not always reasonable, as it is likely that patients with a high optimal threshold have different characteristics to those with a low optimal threshold. However, since we do not have the individual thresholds across the population, and instead have a distribution, this assumption needs to be made.

**Key Assumption 1.3.1)** $f\left( X \right),Y\perp A,B,C,D$

We use this assumption to remove the conditionals of the utility:

$$U\left( f(x),a,b,c,d \right)=aP\left( y=1,f\left( x \right)>t \right)+bP\left( y=0,f\left( x \right)>t \right)$$

$$+cP\left( y=1,f\left( x \right)<t \right)+dP\left( y=0,f\left( x \right)<t \right)$$

We want to approximate the expected estimate across the population.

$$E_{f(x),a,b,c,d\sim X,A,B,C,D}U=\int_{f(X),A,B,C,D} p(f(x),a,b,c,d)(aP\left( y=1,f\left( x \right)>t \right)+bP\left( y=0,f\left( x \right)>t \right)+cP\left( y=1,f\left( x \right)<t \right)+dP\left( y=0,f\left( x \right)<t \right))\mathbf{d}f\mathbf{(}x)\mathbf{d}a\mathbf{d}c\mathbf{d}b\mathbf{d}d$$

We have assumed that $f(X)\perp A,B,C,D$, and thus $p\left( f(x),a,b,c,d \right)=p\left( f(x) \right)p(a,b,c,d)$ so:

$$E_{f(x),a,b,c,d\sim f(X),A,B,C,D}U=\int_{A,B,C,D} p(a,b,c,d)(a\int_{f(X)} P\left( y=1,f\left( x \right)>t \right)p\left( f\left( x \right) \right)\mathbf{d}f\left( x \right)+b\int_{f(X)} P\left( y=0,f\left( x \right)>t \right)p\left( f\left( x \right) \right)\mathbf{d}f\left( x \right)+c\int_{f(X)} P\left( y=1,f\left( x \right)<t \right)p\left( f\left( x \right) \right)\mathbf{d}f\left( x \right)+d\int_{f(X)} P\left( y=0,f\left( x \right)<t \right)p\left( f\left( x \right) \right)\mathbf{d}f\left( x \right)\mathbf{)d}a\mathbf{d}b\mathbf{d}c\mathbf{d}d$$

We can substitute each of the integrals over $f(X)$ over their estimate, i.e., the true and false positives and true and false negatives of the entire population.

$$E_{f(x),a,b,c,d\sim X,A,B,C,D}U=\int_{A,B,C,D} p(a,b,c,d)(aTP\left( t^{*} \right)+bFP\left( t^{*} \right)+cFN\left( t^{*} \right)+dFP\left( t^{*} \right)\boldsymbol{)}\mathbf{d}a\mathbf{d}b\mathbf{d}c\mathbf{d}d$$

In order to make the following step, the second key assumption is needed. The integral above is over four degrees of freedom, and could not be solved without knowledge of the distribution $p\left( a,b,c,d \right)$, even if we know or assume $p(t^{*})$.

Instead, we need to be able to take the expected utility over values of $T^{*}$, for this to be possible, the values of $a$, $b$, $c$ and $d$ need to be fully determined by $t^{*}$. In other words, the distribution of the four utilities needs to have a single degree of freedom, which can be considered a latent variable (perhaps, overall health) which needs to have a one-to-one mapping with the optimal threshold $t^{*}=\frac{1}{1+\frac{a-c}{d-b}}$. In a way, since we can always take a linear transformation of the utility in order to get a meaningful net benefit, we could always set $c$ and $d$ to be constant so that the two utilities have at most two degrees of freedom.

**Key Assumption 1.3.2)** There exists four well-defined functions $g_{a}$, $g_{b}$, $g_{c}$ and $g_{d}$ so that $a=g_{a}(t^{*})$, $b=g_{b}(t^{*})$, $c=g_{c}(t^{*})$ and $d=g_{d}(t^{*})$.

In this case, we can instead take the expected value over $T^{*}$, so that we get:

$$E_{f(x),a,b,c,d\sim f(X),A,B,C,D}U=E_{f(x),t^{*}\sim f(X),T^{*}}U=\int_{T^{*}} p\left( t^{*} \right)\left( aTP\left( t^{*} \right)+bFP\left( t^{*} \right)+cFN\left( t^{*} \right)+dFP\left( t^{*} \right) \right)\mathbf{d}t^{*}$$

From this point, the derivation is very similar to that of the continuous net benefit:

$$E_{f(x),t^{*}\sim f(X),T^{*}}U=\int_{T^{*}} \frac{p\left( t^{*} \right)}{\frac{1}{a\left( t^{*} \right)-c{(t}^{*})}+\frac{1}{d\left( t^{*} \right)-b(t^{*})}}\left( \frac{1}{t}TP\left( t^{*} \right)-\frac{1}{1-t}FP\left( t^{*} \right) \right)\mathbf{d}t^{*}$$

In this case, the weighting function $\omega_{E}\left( t^{*} \right)=\frac{p(t^{*})}{\frac{1}{a(t^{*})-c(t^{*})}+\frac{1}{b(t^{*})-d(t^{*})}}$ is influenced both by the probability distribution of the optimal thresholds across the population $p(t^{*})$, and by the ‘importance’ of these thresholds, according to the harmonic mean of $a\left( t^{*} \right)-c(t^{*})$ and $d\left( t^{*} \right)-b(t^{*})$.

*Supplementary 1.4: Proof that* $AUNB$ *and* $AUNB_{alt}$ *do not always share rank ordering of models.*

Let’s consider the following expressions:

$$AUNB=\int_{0}^{1} p\left( t^{*} \right)\left( TP\left( t^{*} \right)-\frac{t^{*}}{1-t^{*}}FP\left( t^{*} \right) \right)dt^{*}$$

$$AUNB_{alt}=\int_{0}^{1} p\left( t^{*} \right)\left( \frac{1-t^{*}}{t^{*}}TP\left( t^{*} \right)-FP\left( t^{*} \right) \right)dt^{*}$$

The expressions within the integrals are equivalent when plotting the decision curve as the relate to each other through a monotonic transformation (i.e., $TP\left( t^{*} \right)-\frac{t^{*}}{1-t^{*}}FP\left( t^{*} \right)= \frac{t^{*}}{1-t^{*}}\times\left( \frac{1-t^{*}}{t^{*}}TP\left( t^{*} \right)-FP\left( t^{*} \right) \right)$, where $\frac{t^{*}}{1-t^{*}}$ is always positive in the range $t^{*}\in(0,1)$). Because of this, for two models $M_{1}$ and $M_{2}$ with true and false positives of $TP_{M_{1}}\left( t^{*} \right)$, $TP_{M_{2}}\left( t^{*} \right)$, ${FP}_{M_{1}}\left( t^{*} \right)$, and ${FP}_{M_{2}}\left( t^{*} \right)$ for thresholds $t^{*}\in(0,1)$ then $\forall t^{*}\in(0,1)$ we have:

$$TP_{M_{1}}\left( t^{*} \right)-\frac{t^{*}}{1-t^{*}}FP_{M_{1}}\left( t^{*} \right)>TP_{M_{2}}\left( t^{*} \right)-\frac{t^{*}}{1-t^{*}}FP_{M_{2}}\left( t^{*} \right)$$

$$\Longleftrightarrow$$

$$\frac{1-t^{*}}{t^{*}} TP_{M_{1}}\left( t^{*} \right)-FP_{M_{1}}\left( t^{*} \right)>\frac{1-t^{*}}{t^{*}}TP_{M_{2}}\left( t^{*} \right)-FP_{M_{2}}\left( t^{*} \right)$$

However, we show that this is not the case for the integrals $AUNB$ and $AUNB_{alt}$ through a counterexample. Consider two models $M_{1}:\hat{Y}_{M_{1}}=f(X)$ and $M_{2}:\hat{Y}_{M_{2}}=g(X)$. We consider the performance of both models in a population of three groups, $G_{1}$, $G_{2}$ and $G_{3}$.

Individuals in group $G_{1}$ always have $Y_{G_{1}}=1$, $f\left( X_{G_{1}} \right)=0.1$ and $g\left( X_{G_{1}} \right)=0.05$.

Individuals in group $G_{2}$ always have $Y_{G_{2}}=1$, $f\left( X_{G_{2}} \right)=0.12$ and $g\left( X_{G_{2}} \right)=0.2$.

Individuals in group $G_{3}$ always have $Y_{G_{3}}=0$, $f\left( X_{G_{3}} \right)=0.02$ and $g\left( X_{G_{3}} \right)=0.02$.

The groups each constitute a third of the population. Across the three groups, the optimal threshold of individuals is uniformly distributed between 1% and 21%, so that $p\left( t^{*} \right)=5$ for $0.01<t^{*}<0.21$.

To calculate $AUNB$ and $AUNB_{alt}$ for the two models, we first calculate their true positive and false positive proportions in the population of interest:

For Model $M_{1}$, we have true positives $TP_{M_{1}}(t^{*})=\frac{2}{3}$ for $t^{*}<0.1$, $TP_{M_{1}}\left( t^{*} \right)=\frac{1}{3}$ for $0.1<t^{*}<0.12$ and $TP_{M_{1}}\left( t^{*} \right)=0$ for $t^{*}>0.12$, and false positives $FP_{M_{1}}\left( t^{*} \right)=\frac{1}{3}$ for $t^{*}<0.02$ and $FP_{M_{1}}\left( t^{*} \right)=0$ for $t^{*}>0.02$.

For Model $M_{2}$, we have true positives $TP_{M_{2}}(t^{*})=\frac{2}{3}$ for $t^{*}<0.05$, $TP_{M_{2}}\left( t^{*} \right)=\frac{1}{3}$ for $0.05<t^{*}<0.2$ and $TP_{M_{2}}\left( t^{*} \right)=0$ for $t^{*}>0.2$, and false positives $FP_{M_{2}}\left( t^{*} \right)=\frac{1}{3}$ for $t^{*}<0.02$ and $FP_{M_{2}}\left( t^{*} \right)=0$ for $t^{*}>0.02$.

Let’s use this to calculate the $AUNB$ of both models.

$$AUNB_{M_{1}}=\int_{0}^{1} p\left( t^{*} \right)\left( TP_{M_{1}}\left( t^{*} \right)-\frac{t^{*}}{1-t^{*}}FP_{M_{1}}\left( t^{*} \right) \right)dt^{*}$$

$$=5\int_{0.01}^{0.21} TP_{M_{1}}\left( t^{*} \right)dt^{*}-5\int_{0.01}^{0.21} \frac{t^{*}}{1-t^{*}}FP_{M_{1}}\left( t^{*} \right)dt^{*}$$

$$=5\left[ \int_{0.01}^{0.1} \frac{2}{3}dt^{*}+\int_{0.1}^{0.12} \frac{1}{3}dt^{*} \right]-5\int_{0.01}^{0.02} \frac{1}{3}\frac{t^{*}}{1-t^{*}}dt^{*}$$

$$=\frac{10}{3}\left( 0.1-0.01 \right)+\frac{5}{3}\left( 0.12-0.1 \right)-\frac{5}{3}\left[ -t^{*}-\log\left( 1-t^{*} \right) \right|_{0.01}^{0.02}$$

$$\cong0.3331$$

$$AUNB_{M_{2}}=\int_{0}^{1} p\left( t^{*} \right)\left( TP_{M_{2}}\left( t^{*} \right)-\frac{t^{*}}{1-t^{*}}FP_{M_{2}}\left( t^{*} \right) \right)dt^{*}$$

$$=5\int_{0.01}^{0.21} TP_{M_{2}}\left( t^{*} \right)dt^{*}-5\int_{0.01}^{0.21} \frac{t^{*}}{1-t^{*}}FP_{M_{2}}\left( t^{*} \right)dt^{*}$$

$$=5\left[ \int_{0.01}^{0.05} \frac{2}{3}dt^{*}+\int_{0.05}^{0.2} \frac{1}{3}dt^{*} \right]-5\int_{0.01}^{0.02} \frac{1}{3}\frac{t^{*}}{1-t^{*}}dt^{*}$$

$$=\frac{10}{3}\left( 0.05-0.01 \right)+\frac{5}{3}\left( 0.2-0.05 \right)-\frac{5}{3}\left[ -t^{*}-\log\left( 1-t^{*} \right) \right|_{0.01}^{0.02}$$

$$\cong0.3831$$

According to the $AUNB$, model $M_{2}$ is superior to model $M_{1}$. If we check the models’ $AUNB_{alt}$:

$$AUN{B_{alt}}_{M_{1}}=\int_{0}^{1} p\left( t^{*} \right)\left( \frac{1-t^{*}}{t^{*}}TP_{M_{1}}\left( t^{*} \right)-FP_{M_{1}}\left( t^{*} \right) \right)dt^{*}$$

$$=5\int_{0.01}^{0.21} \frac{1-t^{*}}{t^{*}}TP_{M_{1}}\left( t^{*} \right)dt^{*}-5\int_{0.01}^{0.21} FP_{M_{1}}\left( t^{*} \right)dt^{*}$$

$$=\frac{10}{3}\left( \log\left( t^{*} \right)-t^{*} \right|_{0.01}^{0.1}+\frac{5}{3}\left( \log\left( t^{*} \right)-t^{*} \right|_{0.1}^{0.12}-\frac{5}{3}\left( 0.02-0.01 \right)$$

$$\cong7.6292$$

$$AUN{B_{alt}}_{M_{2}}=\int_{0}^{1} p\left( t^{*} \right)\left( \frac{1-t^{*}}{t^{*}}TP_{M_{2}}\left( t^{*} \right)-FP_{M_{2}}\left( t^{*} \right) \right)dt^{*}$$

$$=5\int_{0.01}^{0.21} \frac{1-t^{*}}{t^{*}}TP_{M_{2}}\left( t^{*} \right)dt^{*}-5\int_{0.01}^{0.21} FP_{M_{2}}\left( t^{*} \right)dt^{*}$$

$$=\frac{10}{3}\left( \log\left( t^{*} \right)-t^{*} \right|_{0.01}^{0.05}+\frac{5}{3}\left( \log\left( t^{*} \right)-t^{*} \right|_{0.05}^{0.20}-\frac{5}{3}\left( 0.02-0.01 \right)$$

$$\cong7.2753$$

Thus, according to the $AUNB_{alt}$, model $M_{1}$ is superior to model $M_{2}$. Thus, we have proved by counter-example that $AUNB$ and $AUNB_{alt}$ do not always agree on which model is superior.

Intuitively, we can see this by thinking about how the $AUNB_{alt}$ is equivalent to the $AUNB$ (up to a constant factor) if we change the optimal threshold distribution from $p(t^{*}$) to $\frac{1-t^{*}}{t^{*}}\times p(t^{*})$. We would expect a different ‘shape’ of optimal threshold distribution to rank models differently, which is why using the $AUNB$ and the $AUNB_{alt}$ can lead to opposite conclusions about which models are more beneficial.
