# Supplementary material for: The continuous net benefit: assessing the clinical utility of prediction models when informing a continuum of decisions
Source: Diagn Progn Res. 2026 Feb 17;10:8. doi: 10.1186/s41512-026-00224-z (PMC12911006; doi:10.1186/s41512-026-00224-z)
Supplement: Supplementary file 4 — Additional file 4. (.R): Full code (in R) of the model development and validation presented in the Results section [file 41512_2026_224_MOESM4_ESM.docx]

*Supplementary 3: Development and validation of the cardiovascular risk prediction models*

In this supplementary, we detail the development and validation of the two cardiovascular models presented in the examples of this study. This supplementary loosely follows the Methods section of the TRIPOD reporting checklist (<https://doi.org/10.1186/s12916-014-0241-z>). Full code for this analysis is included in Supplementary 4.

*S3.1. Sources of data and participants*

The development and validation data comprises individuals from the Framingham Heart Study, a prospective observational study in which individuals had baseline predictors recorded and were followed over 24 years for outcomes related to cardiovascular disease. It is sourced from the riskCommunicator R package (10.32614/CRAN.package.riskCommunicator).

All individuals were considered eligible for the training and internal validation of the model, as long as they had 10 years of follow-up (individuals who died or were otherwise censored before this were excluded from the study). As the Framingham Heart Study ran from 1956 to 1968, no patient in the data was prescribed statins.

*S3.2. Outcome and predictors*

The outcome was defined as the development of a new cardiovascular disease event within 10 years after baseline. The predictors used were: age at baseline, sex, current smoking status, education, and body-mass index, serum total cholesterol, systolic blood pressure, prevalent diabetes, use of anti-hypertensive medication, previous cardiovascular disease, prevalent hypertension, and casual serum glucose.

*S3.3. Sample size and missing data*

Since the aim of this analysis is not to actually develop and validate a potentially useful prediction model, but to showcase how to calculate and present the net benefit, we did not carry out any sample size calculations. The Framingham Heart Study dataset has been used frequently for the development of clinical prediction models in a didactic environment.

Single imputation, using the mice package in R (10.32614/CRAN.package.mice), was used to impute any missing data, with 30 iterations used.

In order to account for the possibility of dependent censoring biasing the model’s predictions and the estimation of the performance metrics, inverse probability of censoring weighting (IPCW, <https://doi.org/10.1177/0962280216628900>), was used in order to up-weight individuals more likely to have been censored by 10 years of follow-up. This weighting was applied both when fitting the models and when calculating all the presented performance metrics. The issue of competing risks was not explored in this example. The full set of predictors used by the models was also used to build the propensity score model.

*S3.4. Statistical analysis methods*

For models/policies were considered:

1. Full Model: A logistic regression which uses all available predictors.
2. Dichotomised Model: A dichotomised version of the Full Model, in which all individuals with scores above 10% are treated as high-risk, and those below are treated as low-risk, for all interventions informed by the model.
3. Small Model: A logistic regression which uses a smaller, easy to collect, set of predictors (age, sex, education, body-mass index, and smoking status).
4. Marker-based Policy: A policy which treats an individual as high-risk if they either are on anti-hypertensives, have hypertension, have had previous history of cardiovascular disease, or have a serum total cholesterol reading of over 300 mg/dL.

The continuous net benefit was calculated as outlined in the main manuscript with the different weighting functions outlined in the examples. The optimism and 95% confidence intervals of the continuous net benefit was calculated in the same way. Optimism was corrected through 500 bootstraps, which were used to calculate the difference between the apparent and actual performance of the models. 95% confidence intervals were also calculated through 500 bootstraps.

Supplementary 3, Table 1: Net benefit and weighting function values for Example 1, considering four policies, as well as treat-none and treat-all policies, for cardiovascular risk prognosis for multiple interventions. The net benefit is given in true positives per 100 individuals.

| Threshold | Treat None | Treat All | Full Model | Dichotomised Model | Small Model | Marker-based Policy | Importance Weights |
| --- | --- | --- | --- | --- | --- | --- | --- |
| 0.015 | 0 | 11.75 | 11.84 | 10.08 | 11.79 | 9.36 | 0.00 |
| 0.020 | 0 | 11.30 | 11.51 | 9.95 | 11.38 | 9.22 | 0.00 |
| 0.025 | 0 | 10.85 | 11.16 | 9.82 | 10.94 | 9.07 | 0.00 |
| 0.030 | 0 | 10.39 | 10.84 | 9.68 | 10.56 | 8.92 | 0.01 |
| 0.035 | 0 | 9.92 | 10.46 | 9.55 | 10.22 | 8.77 | 0.02 |
| 0.040 | 0 | 9.45 | 10.12 | 9.41 | 9.85 | 8.62 | 0.04 |
| 0.045 | 0 | 8.98 | 9.86 | 9.27 | 9.50 | 8.47 | 0.09 |
| 0.050 | 0 | 8.50 | 9.62 | 9.13 | 9.25 | 8.31 | 0.18 |
| 0.055 | 0 | 8.01 | 9.44 | 8.99 | 8.89 | 8.16 | 0.32 |
| 0.060 | 0 | 7.53 | 9.16 | 8.85 | 8.67 | 8.00 | 0.54 |
| 0.065 | 0 | 7.03 | 8.92 | 8.70 | 8.40 | 7.84 | 0.86 |
| 0.070 | 0 | 6.53 | 8.62 | 8.56 | 8.09 | 7.68 | 1.30 |
| 0.075 | 0 | 6.03 | 8.49 | 8.41 | 7.70 | 7.52 | 1.83 |
| 0.080 | 0 | 5.52 | 8.28 | 8.26 | 7.30 | 7.35 | 2.42 |
| 0.085 | 0 | 5.00 | 8.14 | 8.11 | 7.05 | 7.18 | 3.01 |
| 0.090 | 0 | 4.48 | 7.90 | 7.96 | 6.90 | 7.02 | 3.52 |
| 0.095 | 0 | 3.95 | 7.87 | 7.80 | 6.70 | 6.85 | 3.87 |
| 0.100 | 0 | 3.42 | 7.65 | 7.65 | 6.43 | 6.67 | 3.99 |
| 0.105 | 0 | 2.88 | 7.47 | 7.49 | 6.20 | 6.50 | 0.00 |
| 0.110 | 0 | 2.33 | 7.33 | 7.33 | 5.86 | 6.32 | 0.00 |
| 0.115 | 0 | 1.78 | 7.14 | 7.17 | 5.64 | 6.15 | 0.00 |
| 0.120 | 0 | 1.22 | 7.02 | 7.01 | 5.33 | 5.97 | 0.00 |

Supplementary 3, Table 2: Net benefit (evaluated at the 10% threshold) and continuous net benefit (with the weighting function specified in Supplementary 3, Table 2), in true positives per 100 patients, corresponding to the values presented in Figure 1b of the manuscript.

| Model | Statins (Net Benefit at 10%) | Overall Management (Continuous Net Benefit with Weighting Function) |
| --- | --- | --- |
| Treat No One | 0.00 (0.00–0.00) | 0.00 (0.00–0.00) |
| Treat All | 3.33 (2.37–4.28) | 5.07 (4.13–6.01) |
| Full Model | 7.69 (6.78–8.50) | 8.26 (7.36–9.13) |
| Dichotomised Model | 7.69 (6.78–8.50) | 8.20 (7.31–9.01) |
| Small Model | 6.47 (5.56–7.33) | 7.32 (6.38–8.19) |
| Marker-based Policy | 6.71 (5.84–7.60) | 7.27 (6.40–8.15) |

Supplementary 3, Table 3: Net benefit and weighting function values for Example 2, considering four policies, as well as treat-none and treat-all policies, for cardiovascular risk prognosis for one intervention with multiple threshold. The net benefit is given in true positives per 100 individuals.

| Threshold | Treat None | Treat All | Full Model | Dichotomised Model | Small Model | Marker-based Policy | Importance Weights |
| --- | --- | --- | --- | --- | --- | --- | --- |
| 0.020 | 0 | 11.22 | 11.51 | 9.93 | 11.38 | 9.22 | 0.00 |
| 0.030 | 0 | 10.30 | 10.85 | 9.66 | 10.56 | 8.92 | 0.00 |
| 0.040 | 0 | 9.37 | 10.13 | 9.39 | 9.84 | 8.62 | 0.01 |
| 0.050 | 0 | 8.41 | 9.62 | 9.11 | 9.25 | 8.31 | 0.07 |
| 0.060 | 0 | 7.44 | 9.17 | 8.83 | 8.66 | 8.00 | 0.29 |
| 0.070 | 0 | 6.44 | 8.64 | 8.54 | 8.09 | 7.68 | 0.70 |
| 0.080 | 0 | 5.43 | 8.28 | 8.24 | 7.29 | 7.35 | 1.23 |
| 0.090 | 0 | 4.39 | 7.89 | 7.94 | 6.92 | 7.02 | 1.70 |
| 0.100 | 0 | 3.33 | 7.63 | 7.63 | 6.46 | 6.67 | 1.99 |
| 0.110 | 0 | 2.24 | 7.31 | 7.31 | 5.91 | 6.32 | 2.06 |
| 0.120 | 0 | 1.13 | 7.01 | 6.99 | 5.38 | 5.97 | 1.95 |
| 0.130 | 0 | -0.01 | 6.56 | 6.66 | 4.96 | 5.60 | 1.72 |
| 0.140 | 0 | -1.17 | 6.35 | 6.32 | 4.47 | 5.23 | 1.43 |
| 0.150 | 0 | -2.36 | 6.13 | 5.97 | 4.21 | 4.84 | 1.14 |
| 0.160 | 0 | -3.58 | 5.86 | 5.62 | 3.74 | 4.45 | 0.88 |
| 0.170 | 0 | -4.83 | 5.42 | 5.25 | 3.46 | 4.05 | 0.66 |
| 0.180 | 0 | -6.11 | 5.23 | 4.88 | 3.09 | 3.64 | 0.49 |
| 0.190 | 0 | -7.42 | 5.03 | 4.50 | 2.69 | 3.22 | 0.35 |
| 0.200 | 0 | -8.76 | 5.03 | 4.11 | 2.51 | 2.78 | 0.25 |
| 0.210 | 0 | -10.14 | 4.98 | 3.71 | 2.27 | 2.34 | 0.18 |
| 0.220 | 0 | -11.55 | 4.93 | 3.29 | 2.14 | 1.89 | 0.12 |
| 0.230 | 0 | -13.00 | 4.95 | 2.87 | 1.86 | 1.42 | 0.08 |
| 0.240 | 0 | -14.48 | 4.72 | 2.44 | 1.50 | 0.94 | 0.06 |
| 0.250 | 0 | -16.01 | 4.64 | 1.99 | 1.36 | 0.45 | 0.04 |
| 0.260 | 0 | -17.58 | 4.57 | 1.54 | 1.20 | -0.06 | 0.03 |
| 0.270 | 0 | -19.19 | 4.40 | 1.07 | 0.93 | -0.57 | 0.02 |
| 0.280 | 0 | -20.84 | 4.35 | 0.58 | 0.78 | -1.11 | 0.01 |
| 0.290 | 0 | -22.55 | 4.24 | 0.09 | 0.65 | -1.66 | 0.01 |
| 0.300 | 0 | -24.30 | 4.11 | -0.42 | 0.44 | -2.22 | 0.01 |

Supplementary 3, Table 4: Net benefit (evaluated at the 10% threshold) and continuous net benefit (with the weighting function specified in Supplementary 3, Table 3), in true positives per 100 patients, corresponding to the values presented in Figure 2b of the manuscript.

| Model | Statins (Net Benefit at 10%) | Statins, Distribution (Continuous Net Benefit with Weighting Function) |
| --- | --- | --- |
| Treat No One | 0.00 (0.00–0.00) | 0.00 (0.00–0.00) |
| Treat All | 3.33 (2.37–4.28) | 5.07 (4.13–6.01) |
| Full Model | 7.69 (6.78–8.50) | 8.26 (7.36–9.13) |
| Dichotomised Model | 7.69 (6.78–8.50) | 8.20 (7.31–9.01) |
| Small Model | 6.47 (5.56–7.33) | 7.32 (6.38–8.19) |
| Marker-based Policy | 6.71 (5.84–7.60) | 7.27 (6.40–8.15) |
